# Duration of exclusive breastfeeding may be related to eating behaviour and dietary intake in obesity prone normal weight young children

**DOI:** 10.1371/journal.pone.0200388

**Published:** 2018-07-11

**Authors:** Ina Olmer Specht, Jeanett Friis Rohde, Nanna Julie Olsen, Berit Lilienthal Heitmann

**Affiliations:** 1 The Parker Institute, Research Unit for Dietary Studies, Bispebjerg and Frederiksberg Hospital, Frederiksberg, Denmark; 2 The Boden Institute of Obesity, Nutrition, Exercise & Eating Disorders, The University of Sydney, Sydney, Australia; 3 Department of Public Health, Section for General Medicine, University of Copenhagen, Copenhagen, Denmark; The University of Tokyo, JAPAN

## Abstract

Infants who are breastfed are introduced to a variety of flavours from the maternal milk, and thus the transition from maternal milk to complementary foods may be easier for these children. The aim of this study was to investigate if duration of exclusive breastfeeding was associated with pickiness or dietary intake of vegetables, fruit, starchy foods or sugar sweetened beverages among obesity prone normal weight children aged 2–6 years. This cohort study was based on data from the Healthy Start primary intervention study, the Danish Medical Birth registry and the Danish Health Visitor’s Child Health Database. Infant feeding was registered by health nurses while home-visiting the mother and child up to four times within the first year. Information on eating behaviour and diet intake at age 2–6 years was obtained by parents. Crude and adjusted logistic and general linear regression models were used to investigate associations. A total of 236 children had complete information on all variables. Data showed lower odds of picky eating behaviour when exclusively breastfed until age 4–5 months compared to exclusively breastfed for 0–1 months (OR = 0.35, 95CI = 0.16;0.76, p = 0.008). In the crude analysis only, exclusively breastfed until age 6–10 months was associated with a higher daily intake of vegetables (p = 0.04). This study suggests that exclusive breastfeeding duration seems to influence pickiness and may contribute to facilitate the consumption of more vegetables in later childhood in obesity prone normal weight children.

## Introduction

The many benefits of breastfeeding are well known [1, 2]. As recommended by the World Health Organisation (WHO), six months is the optimal duration of exclusive breastfeeding (no intake of additional food or drink) in relation to optimal growth, health and development of the child [1, 3]. Still, in many European countries only some 50% [4, 5], or even fewer [6, 7], are breastfed until 6 months of age.

Childhood pickiness is prevalent in the Western world with 20–50% picky eaters especially in the age group 2–4 years [8, 9]. Children that tend to have picky eating behaviours often have a lower diet quality [10], eat less fruit and vegetables [11–14] and have higher intakes of sugary foods and drinks [15]. The consequences of pickiness can be malnutrition and may, if prolonged, have an impact on weight development and risk of overweight and obesity [16], even though not all studies have observed this association [13]. The acceptance of healthy food choices later in childhood seems associated with breastfeeding duration independent of socio-economic status (SES) [17]. Several studies have also shown associations between a longer duration of breastfeeding and a lower degree of pickiness in childhood [11, 12, 18, 19], and some studies have suggested six months of exclusive breastfeeding to be a threshold for not developing pickiness [12, 19], which corresponds well with the recommendations by the WHO.

The transition from breast milk to complementary foods seems easier for children being exclusively breastfed compared to formula fed children. One suggested explanation is that a variety of flavours from the maternal diet have been introduced in the breast milk, which are not present in formula, and thus makes it easier for the breastfed infants to accept similar flavoured foods [20–22].

Studies have also shown that children who were breastfed for a short duration or exclusively formula fed tended to eat a less healthy diet in later childhood [23, 24]. Understanding early infant eating behaviours is therefore important for the promotion of healthy childhood eating, including increased fruit and vegetables consumption, especially in a population of children with increased risk of later obesity.

We aimed to investigate if duration of exclusive breastfeeding was associated with pickiness or intake of vegetables, fruit, starchy foods or sugar sweetened beverages in obesity prone normal weight children.

## Materials and methods

This cohort study was based on data from the Healthy Start primary intervention study, data from the Danish Medical Birth registry (MBR) and the Danish Health Visitor’s Child Health Database.

The Healthy Start study was a randomized controlled primary prevention intervention conducted between 2009 and 2011 in 11 municipalities around the greater Copenhagen area [25]. The aim was to prevent overweight in children, who were yet normal weight, but were susceptible to future overweight (obesity prone), as they were all either born with a high birth weight (> 4,000 grams) and/or had a mother who was overweight prior to pregnancy (body mass index (BMI) > 28 kg/m^2^). In addition, a subgroup of children was selected because their mother had a short educational level (≤ 10 years). This intervention aimed in a 15 months period to improve dietary and physical activity behaviours, reduce stress and improve sleep quality and quantity and thereby diminishing the children’s risk of developing childhood obesity. The methodology of the Healthy Start primary intervention study has been described in closer detail elsewhere [25]. From the Healthy Start primary intervention we have data on diet intake and picky eating behaviour at baseline, when the children were 2–6 years. Further, we have data on picky eating behaviour again in the same children 15 months later. The intervention did not show any effects on picky eating behaviour [13], thus we use data on picky eating behaviour from the 15 months follow-up to investigate if eating behaviour changed over time.

We obtained information on maternal smoking during pregnancy, gestational age of birth, birth weight, parity and municipality of residence from the MBR. From the Danish Health Visitor’s Child Health Database we obtained information about breastfeeding duration and number of health nurse visits collected by health visitors. The Database Steering Committee provided the data.

This study was conducted according to the guidelines laid down by the Declaration of Helsinki. The Danish Data Protection Agency approved the usage of the data obtained in the Healthy Start primary prevention intervention (journal number: 2015-41-3937). The Scientific Ethical Committee of the Capital Region in Denmark decided that the project was not a bioethics project, and consequently did not need approval from the Danish Bioethics Committee (journal number H-A-2007-0019). Written informed consent to use the collected data for research purposes was obtained from all parents. The ClinicalTrials.gov identifier for The Health Start study is NCT01583335.

### Study population

The Healthy Start intervention study initially invited 3,058 children, identified in registries, born in 2004–2007 (n: 1,523 intervention group and 1,535 control group) and aged 2–6 years to participate, and about 21% in each group agreed. Thus, the original study population included a total of 543 normal weight obesity prone children with baseline information on among others weight, eating habits, sleep, stress and physical activity [25]. For the present study, children with missing information on the exposure variables (breastfeeding, n = 221) and the outcome variable (pickiness, n = 59) were excluded, and therefore the final study population in the crude analysis consisted of 263 children and 236 children in the analyses adjusted for potential confounders.

When we investigated changes in eating behaviour from baseline and after the intervention we included all children with data on picky eating behaviour at baseline (n = 484).

Children with and without breastfeeding information were not different in regard to pickiness at baseline (chi-square p-value = 0.20).

### Determination of infant feeding

Infant feeding was registered by health nurses while visiting the new mothers when the child was few days old and until the child was approximately 10 months old. They visited the family up to 4 times. All women in Denmark are offered these home-visits free of charge.

The exposure variable was categorized into exclusively breastfed until age 0–1 months, 2–3 months, 4–5 months and 6–10 months. Exclusively breastfed means that the infant did not have an intake of additional foods or drinks. Thus, children who were formula fed from birth would be in the group 0–1 months of exclusively breastfeeding, and children supplemented with formula from e.g. month 3 would be in the group of 2–3. Children starting complementary food at age 6 months would be in the group of 4–5 months of exclusively breastfeeding.

### Determination of eating behaviour

At baseline and follow-up parents completed questionnaires on meal habits and family well-being. Information on pickiness was based on the question *“How would you describe your child’s way of eating*?*”* The parents could answer if they perceived their child as being “*picky*”, “*a little picky”* or “*likes everything*”.

Due to power issues in the analysis of breastfeeding and eating behaviour we cumulated children with picky eating behaviour and children who were a little picky, to get a dichotomised outcome variable used in the main analysis (picky/non-picky).

### Dietary measurements

Information on the child’s diet intake was obtained by parents at baseline when the children were 2–6 years of age using a 4-day (Wednesday-Saturday) diet record, applying a picture book as guidance in reporting portion sizes [26]. The software Dankost 3000 was used for nutrient calculation (http://dankost.dk/). Since it was not possible to define food groups using Dankost 3000, this was done manually by studying the list of all food items eaten by the children. A Stata program was then developed by a statistician to extract information and generate food groups (fruit, vegetables, starchy foods and sugar sweetened beverages). Definitions of food groups were based on standards developed by the National Food Institute, Technical University of Denmark [27] and further modified to fit the Danish dietary guidelines and to suit the Healthy Start primary intervention study. Dietary food group information was used to investigate if there were any associations between duration of exclusive breastfeeding and intake of vegetable, fruit, starchy foods and sugar sweetened beverages. Fruit was defined as: fresh, canned and frozen (excluding jam, fruit juice, dried fruit and fruit products with added sugar), vegetables as: fresh, canned and frozen (excluding fried onion, ketchup, pickles and potatoes), starchy foods as: rice, pasta or potatoes, and sugar sweetened beverages as: all beverage with added sugar. All food groups were presented in g/day.

### Statistical methods

Logistic regression was used to analyse crude and adjusted associations between infant feeding and eating behaviour in early childhood.

In analysis investigating dietary intake we used general linear models with dietary intake as the continuous outcome variable in grams per day, and breastfeeding duration.

In the multiple logistic regression models we adjusted for confounding by *a priori* known and potential confounding variables, namely smoking during pregnancy (no/yes), parity (1 child/ 2 children/ ≥3 children), problems establishing breastfeeding (yes/no), maternal age at pregnancy (tertiles: 19–30 / 30-33/ 34–46 years) [10] and maternal municipality of residence at date of giving birth.

On pickiness and dietary groups, we examined interactive effects of duration of breastfeeding and maternal education (no academic/ up to bachelor degree/ academic degree), and child’s age at baseline (2–3 / 3–5 / 5–6 years).

We further investigated changes in pickiness from baseline to follow-up using McNiemer’s test for symmetry.

Statistical analysis of the data was performed with SAS Enterprise Guide software, version 7.1 for Windows (SAS Institute Inc., NC, USA).

## Results

As shown in Table 1, the median birth weight of 4150 (1800–5450) reflects the inclusion criteria well. 20.2% of the children were only exclusively breastfed until age 0–1 month, and 14.8% were exclusively breastfeed until age 6–10 months. The majority, 52.9% of the children, were exclusively breastfeed until age 4–5 months. Picky eating behaviour was observed in 16.0% of the children and 46.4% were a little picky at baseline, when they were on average 3.9 (2.1–6.0) years old (Table 1).

**Table 1:** Characteristics of the mother and child.

*Child characteristics*	n	Median (range)	%
Birth weight	263	4150 (1800–5450)	
Gestational age at birth	263	284 (230–299)	
Age in days at:			
first health nurse visit	244	9 (0–43)	
second health nurse visit	198	69 (8–144)	
third health nurse visit	228	133 (92–252)	
fourth health nurse visit	216	258 (182–311)	
Mothers with 4 health nurse visits	225		85.6
Exclusively breastfeeding duration (months)	263	4 (0–10)	
Exclusively breastfed at age:			
0–1 months	53		20.2
2–3 months	32		12.2
4–5 months	139		52.9
6–10 months	39		14.8
Exclusively formula fed at first visit	9		3.8
Exclusively formula fed at second visit	23		10.4
Exclusively formula fed at both visits	6		2.9
Problems establishing breastfeeding	73		29.4
Age in years at baseline	263	3.9 (2.1–6.0)	
Age in years at follow-up	174	5.3 (3.2–7.3)	
Child’s Body Mass Index (kg/m2) ^a^	263	16 (12–18)	
Picky eater^a^	42		16.0
A little picky eater^a^	122		46.4
Likes almost everything^a^	99		37.6
*Maternal characteristics*			
Maternal age at pregnancy^b^ (yrs)	263	32.3 (21.6–42.8)	
Parity^b^:			
1 child	101		39.3
2 children	121		47.1
≥3 children	35		13.6
Smoking during pregnancy^b^	16		6.1
Maternal education^a^:			
No academic education	65		25.9
Bachelor degree	120		47.8
Master degree	66		26.3

^a^ At baseline when the children were 2–6 years of age.

^b^ Data obtained from the Medical Birth Registry

We investigated changes in picky eating behaviour from baseline to follow-up, e.g. changes over a period of 15 months. Overall the children did not change picky eating behaviour which was also confirmed by a McNiemer’s test for symmetry of 2.55 between baseline and follow-up eating behaviour, with a corresponding p-value of 0.20 (data not shown). In total 41% of the data was missing at follow-up, and thus no further analyses were made in this data.

When investigating the association between duration of exclusive breastfeeding and pickiness, we observed a lower odds ratio (OR) of picky eating behaviour among those exclusively breastfed until age 4–5 months and those exclusively breastfeed for 0–1 month in both crude (OR_crude_ = 0.51 95CI = 0.27;0.98, p = 0.04) and adjusted analyses (OR_adjusted_ = 0.35, 95CI = 0.16;0.76, p = 0.008) (Table 2).

**Table 2:** Picky eating behaviour by infant exclusively breastfeeding.

Exclusively breastfed in months	n	OR_crd_	95% CI	*P*-value	OR_adj._	95% CI	*P*-value
0–1	53	1	-	-	1	-	-
2–3	32	0.71	0.29;1.73	0.45	0.43	0.15;1.20	0.11
4–5	**139**	**0.51**	**0.27;0.98**	**0.04**	**0.35**	**0.16;0.76**	**0.008**
6–10	39	0.58	0.23;1.36	0.21	0.51	0.19;1.37	0.18

Adjusted for parity, smoking during pregnancy, maternal age at pregnancy, problems establishing breastfeeding, and maternal municipality at time of birth.

We observed a higher daily intake of vegetables for those breastfed until age 6–10 months compared to those breastfed for 0–1 month, but this was statistically significant only in the crude analysis (Table 3). No associations between breastfeeding duration and fruit, starchy foods or sugar sweetened beverages were observed in either crude or adjusted analyses (Table 3).

**Table 3:** Vegetables, fruit, starch and sugar sweetened beverages intake pr day in relation to exclusively breastfeeding duration.

		Vegetables g/day					Fruit g/day					Starchy foods g/day					Sugar sweetened beverages g/day				
Exclusively breastfed in months	n	Mean	Mean _adj._	95%CI	*P*_crude_	*P*_adj._	Mean	Mean _adj._	95%CI	*P*_crude_	*P*_adj._	Mean	Mean _adj._	95%CI	*P*_crude_	*P*_adj._	Mean	Mean _adj._	95%CI	*P*_crude_	*P*_adj._
0–1	53	81.7	90.0	(6.3;119.6)	(ref)	(ref)	91.3	92.2	(73.3;111.3)	(ref)	(ref)	51.9	54.3	(43.1;65.4)	(ref)	(ref)	81.5	89.5	(60.9;118.1)	(ref)	(ref)
2–3	32	89.0	98.1	(64.4;131.8)	0.63	0.63	85.0	87.9	(63.7;112.0)	0.64	0.77	61.1	59.8	(45.6;74.0)	0.24	0.52	64.0	67.3	(30.8;103.9)	0.38	0.33
4–5	139	99.1	102.0	(75.5;128.6)	0.11	0.33	89.0	95.2	(81.2;109.2)	0.81	0.78	56.8	54.4	(46.1;62.6)	0.38	0.99	73.9	81.5	(60.4;102.7)	0.50	0.63
6–10	36	**117.3**	113.1	(79.3;147.0)	**0.01**	0.15	108.7	106.7	(83.6;129.9)	0.17	0.30	57.1	54.8	(41.2;68.5)	0.48	0.94	48.4	55.2	(20.2;90.2)	0.08	0.11

Adjusted for parity, smoking during pregnancy, maternal age at pregnancy, problems establishing breastfeeding, and maternal municipality at time of birth.

No interactions between maternal education, age of the child at baseline or pickiness and duration of breastfeeding and dietary intake were observed (data not shown).

## Discussion

Food preferences by children are initiated early in life, partially by genetics but also by infant feeding and the family environment [28, 29]. Understanding these factors may be important to obtain a healthy lifestyle especially in children with increased risk of obesity. In this study we investigated if exclusive breastfeeding duration was associated with pickiness or dietary intake in early childhood among normal weight children with increased risk of obesity.

In our study population children who were breastfed exclusively until age 4–5 months were less likely to be picky compared to children breastfed exclusively for 0–1 month only, regardless of maternal education level. Children exclusively breastfed until end of 5 months of age fulfilled the breastfeeding recommendations by the WHO, of which complementary food should be given from the beginning of the 6^th^ month of age [1]. Other studies have also confirmed that breastfeeding for the first 6 months (corresponding to 5 months of age) reduced the odds of a picky eating behaviour [12, 19]. It has been suggested that children who were breastfed and got complementary food from 4–6 months of age, may be less picky in childhood, because they are exposed to more flavours early in life from both maternal diet during breastfeeding and complementary food [30, 31]. Children exclusively breastfed for 6–10 months were not less picky compared to children breastfed for 0–1 months in our study. These children are exposed to additional flavours from complementary food later in infancy, which might be the reason for the lack of association. The children in our study who were not exclusively breastfed might have been partially breastfed and not exclusively formula fed, thus they might have received some flavours from breast milk or from complementary foods.

Despite our results showed that children breastfed until age 4–5 months were less picky we did not show that this group had a different intake of vegetables, fruit, starchy foods or sugar sweetened beverages than children breastfed for 0–1 month. An earlier study showed greater enjoyment of carrots among children whose mothers ate carrots while breastfeeding compared to children whose mothers did not, however the amount of carrot ate by the two child groups were the same [22]. We only had information on exclusive formula feeding in the two first health nurse visits, of which only 2.9% were bottle fed at both visits, thus we were not able to investigate this group separately. One previous study showed that infants increased their intake of mashed beans after 8 days of exposure, regardless if they were breast or formula fed [32]. However, other studies have shown benefits of breastfeeding duration on later childhood dietary intake [24, 33–35]. Although we did not show significant associations in the adjusted analysis of duration of breastfeeding and grams of vegetables consumed per day, we did show a tendency of higher vegetable consumption corresponding to more months of exclusively breastfeeding. We attribute the lack of significance to the limited sample size.

Picky eating behaviour has been associated with obesity if prolonged [24]. Fortunately, we showed in a previous study in the same study population, that energy intake from unhealthy foods was similar for picky and non-picky children [13]. This is especially fortunate since the children in the present study were all in a higher risk of developing obesity. However, the long-term consequences of picky eating among obesity prone children is unknown, and thus, exclusive breastfeeding, as recommended by the WHO, should be encouraged in this population and in populations where duration of breastfeeding in short. The children in this study were normal weight at baseline but selected according to their increased risk of obesity. They were either born by an obese mother, came from a low SES family or had a high birth weight according to gestational age, since these factors increase the risk of obesity in later life [36–38]. We did not select children specifically by low birth weight, although low birth weight and subsequent rapid growth are additional factors predisposing children to obesity [38]. In this regard, a recent review on birth weight concluded that low birth weight was not associated with a higher energy intake or eating behaviours in later childhood [39].

An advantage of this study is that breastfeeding data was collected by health nurses while visiting the mothers at their private home and was thus not retrospectively collected or self-reported data. A total of 85.6% of the mothers had four visits by the health nurse up to the child was in average 9 months of age, thus recall bias is not an issue in this study. At the health nurse visits one of the main focus areas is breastfeeding, and these frequent visits, might encourage women to continue breastfeeding and help women with difficulties in initiating and maintaining breastfeeding. This is most likely the reason for the high proportion of children exclusively breastfed in Denmark [40] compared to other Western populations [19, 41].

This study also had some limitations in the form of a somewhat small sample size due to missing observations in the breastfeeding variable (n = 221), however we showed that the children with and without missing observations did not differ with respect to picky eating behaviour. The breastfeeding information was collected by the health nurses in their daily work and not as part of a research project, thus on a busy day the nurses might have forgotten to report the breastfeeding. To gain power in the analysis we cumulated children with picky eating behaviour and children who were a little picky into a dichotomised outcome variable. If, instead of using this dichotomised variable, we use the three levels outcome variable we would get cells in the nominal logistic regression model with as few as 7 observations, and hence these analyses could not be performed. However, the dichotomisation may also have resulted in some misclassification that would tend to attenuate our observed relations rather than inflate them, thus giving credit to the significant findings we observed. Another potential limitation is the use of a simple question to measure pickiness rather than multi-item validated scales. However, the fact that we found borderline significant associations given the use of such more crude measure again suggests that our results are real and attenuated rather than inflated. The parents self-reported the child’s eating behaviour and differences in how parents define pickiness may exist. Also, this potential misclassification introduced by using this more crude information may potentially have led to attenuation of our observed associations. In addition, we showed that eating behaviour did not change over a period of 15 months thus we believe that the outcome measure was robust in our population. Unfortunately, we did not have information on maternal SES, maternal income or maternal education at the time of birth. This is a potential confounder since children of parents with high education level or SES have a healthier lifestyle [42]. As proxies for SES in the multiple analyses we included municipality, since the residence of each of the 11 included municipalities have different SES; age of the mother, since young mothers often tend towards a lower SES than older mothers [43]; and parity, since mothers with few children might be associated with a higher SES [43]. We had information on maternal education at baseline when the children were 2–6 years of age, this potential mediator was included in the interaction analysis, but did not influence our results.

## Conclusion

This study found that breastfeeding duration influenced pickiness in young childhood, and potentially facilitate the consumption of more vegetables in later childhood. These results are in agreement with results from previous studies among healthy children, but to our knowledge our study is the first to provide results among obesity prone normal weight children.

## References

[1] Kramer MS, Kakuma R. The Optimal Duration of Exclusive Breastfeeding—A systematic review Switzerland: World Health Organisation, Health DoNf, (NHD) aD; 2001.

[2] Oddy WH. Breastfeeding, Childhood Asthma, and Allergic Disease. Annals of nutrition & metabolism. 2017;70 Suppl 2:26–36. Epub 2017/05/19. doi: 10.1159/000457920 . 28521318 10.1159/000457920

[3] Infant and young child feeding: model chapter for textbooks for medical students and allied health professionals France: WHO Library Cataloguing-in-Publication Data, 2009 978 92 4 159749 4. 23905206

[4] Rodriguez-Perez MJ, Alvarez-Vazquez E, Medina-Pomares J, Velicia-Penas CV, Cal-Conde A, Goicoechea-Castano A, et al [Prevalence of BreastFeeding in a Galician Health Area, Spain]. Revista espanola de salud publica. 2017;91 Epub 2017/02/10. . 28181988 PMC11587300

[5] Weissenborn A, Abou-Dakn M, Bergmann R, Both D, Gresens R, Hahn B, et al [Breastfeeding Rates and Duration in Germany—A Systematic Review]. Gesundheitswesen. 2016;78(11):695–707. Epub 2015/09/04. doi: 10.1055/s-0035-1555946 . 26335658 10.1055/s-0035-1555946

[6] Tarrant RC, Kearney JM. Session 1: Public health nutrition. Breast-feeding practices in Ireland. The Proceedings of the Nutrition Society. 2008;67(4):371–80. Epub 2008/08/22. doi: 10.1017/s0029665108008665 . 18715521 10.1017/S0029665108008665

[7] Callen J, Pinelli J. Incidence and duration of breastfeeding for term infants in Canada, United States, Europe, and Australia: a literature review. Birth (Berkeley, Calif). 2004;31(4):285–92. Epub 2004/11/30. doi: 10.1111/j.0730-7659.2004.00321.x . 15566341 10.1111/j.0730-7659.2004.00321.x

[8] Carruth BR, Ziegler PJ, Gordon A, Barr SI. Prevalence of picky eaters among infants and toddlers and their caregivers' decisions about offering a new food. Journal of the American Dietetic Association. 2004;104(1 Suppl 1):s57–64. Epub 2004/01/01. doi: 10.1016/j.jada.2003.10.024 . 14702019 10.1016/j.jada.2003.10.024

[9] Cole NC, An R, Lee SY, Donovan SM. Correlates of picky eating and food neophobia in young children: a systematic review and meta-analysis. Nutrition reviews. 2017;75(7):516–32. Epub 2017/05/24. doi: 10.1093/nutrit/nux024 . 28535257 10.1093/nutrit/nux024

[10] Byrne R, Yeo MEJ, Mallan K, Magarey A, Daniels L. Is higher formula intake and limited dietary diversity in Australian children at 14 months of age associated with dietary quality at 24 months? Appetite. 2018;120:240–5. Epub 2017/09/14. doi: 10.1016/j.appet.2017.09.002 . 28899651 10.1016/j.appet.2017.09.002

[11] Rossbach S, Foterek K, Schmidt I, Hilbig A, Alexy U. Food neophobia in German adolescents: Determinants and association with dietary habits. Appetite. 2016;101:184–91. Epub 2016/03/02. doi: 10.1016/j.appet.2016.02.159 . 26928790 10.1016/j.appet.2016.02.159

[12] Galloway AT, Lee Y, Birch LL. Predictors and consequences of food neophobia and pickiness in young girls. Journal of the American Dietetic Association. 2003;103(6):692–8. Epub 2003/06/05. doi: 10.1053/jada.2003.50134 ; PubMed Central PMCID: PMCPMC2532522. 12778039 10.1053/jada.2003.50134 PMC2532522

[13] Rohde JF, Handel MN, Stougaard M, Olsen NJ, Traerup M, Mortensen EL, et al Relationship between pickiness and subsequent development in body mass index and diet intake in obesity prone normal weight preschool children. PLoS One. 2017;12(3):e0172772 Epub 2017/03/16. doi: 10.1371/journal.pone.0172772 ; PubMed Central PMCID: PMCPMC5351873. 28296896 10.1371/journal.pone.0172772 PMC5351873

[14] Cooke L, Carnell S, Wardle J. Food neophobia and mealtime food consumption in 4–5 year old children. The international journal of behavioral nutrition and physical activity. 2006;3:14 Epub 2006/07/11. doi: 10.1186/1479-5868-3-14 ; PubMed Central PMCID: PMCPMC1557859. 16824218 10.1186/1479-5868-3-14 PMC1557859

[15] Taylor CM, Northstone K, Wernimont SM, Emmett PM. Macro- and micronutrient intakes in picky eaters: a cause for concern? The American journal of clinical nutrition. 2016;104(6):1647–56. Epub 2016/12/10. doi: 10.3945/ajcn.116.137356 ; PubMed Central PMCID: PMCPMC5118732. 27935522 10.3945/ajcn.116.137356 PMC5118732

[16] Finistrella V, Manco M, Ferrara A, Rustico C, Presaghi F, Morino G. Cross-sectional exploration of maternal reports of food neophobia and pickiness in preschooler-mother dyads. Journal of the American College of Nutrition. 2012;31(3):152–9. Epub 2012/12/04. . 23204151 10.1080/07315724.2012.10720022

[17] Jones L, Moschonis G, Oliveira A, de Lauzon-Guillain B, Manios Y, Xepapadaki P, et al The influence of early feeding practices on healthy diet variety score among pre-school children in four European birth cohorts. Public health nutrition. 2015;18(10):1774–84. Epub 2014/11/21. doi: 10.1017/S1368980014002390 . 25409628 10.1017/S1368980014002390 PMC10271484

[18] Boswell N, Byrne R, Davies PSW. Eating behavior traits associated with demographic variables and implications for obesity outcomes in early childhood. Appetite. 2018;120:482–90. Epub 2017/10/13. doi: 10.1016/j.appet.2017.10.012 . 29024677 10.1016/j.appet.2017.10.012

[19] Shim JE, Kim J, Mathai RA. Associations of infant feeding practices and picky eating behaviors of preschool children. Journal of the American Dietetic Association. 2011;111(9):1363–8. Epub 2011/08/30. doi: 10.1016/j.jada.2011.06.410 . 21872699 10.1016/j.jada.2011.06.410

[20] Mennella JA, Beauchamp GK. Experience with a flavor in mother's milk modifies the infant's acceptance of flavored cereal. Developmental psychobiology. 1999;35(3):197–203. Epub 1999/10/26. . 10531532 10.1002/(sici)1098-2302(199911)35:3<197::aid-dev4>3.0.co;2-j

[21] Mennella JA, Daniels LM, Reiter AR. Learning to like vegetables during breastfeeding: a randomized clinical trial of lactating mothers and infants. The American journal of clinical nutrition. 2017;106(1):67–76. Epub 2017/05/19. doi: 10.3945/ajcn.116.143982 ; PubMed Central PMCID: PMCPMC5486194. 28515063 10.3945/ajcn.116.143982 PMC5486194

[22] Mennella JA, Jagnow CP, Beauchamp GK. Prenatal and postnatal flavor learning by human infants. Pediatrics. 2001;107(6):E88 Epub 2001/06/05. ; PubMed Central PMCID: PMCPMC1351272. 11389286 10.1542/peds.107.6.e88 PMC1351272

[23] Santos LP, Assuncao MC, Matijasevich A, Santos IS, Barros AJ. Dietary intake patterns of children aged 6 years and their association with socioeconomic and demographic characteristics, early feeding practices and body mass index. BMC Public Health. 2016;16(1):1055 Epub 2016/10/08. doi: 10.1186/s12889-016-3725-2 ; PubMed Central PMCID: PMCPMC5052805. 27716197 10.1186/s12889-016-3725-2 PMC5052805

[24] Issanchou S. Determining Factors and Critical Periods in the Formation of Eating Habits: Results from the Habeat Project. Annals of nutrition & metabolism. 2017;70(3):251–6. Epub 2017/04/14. doi: 10.1159/000471514 . 28407627 10.1159/000471514

[25] Olsen NJ, Buch-Andersen T, Handel MN, Ostergaard LM, Pedersen J, Seeger C, et al The Healthy Start project: a randomized, controlled intervention to prevent overweight among normal weight, preschool children at high risk of future overweight. BMC Public Health. 2012;12:590 Epub 2012/08/03. doi: 10.1186/1471-2458-12-590 ; PubMed Central PMCID: PMCPMC3490801. 22852799 10.1186/1471-2458-12-590 PMC3490801

[26] Gondolf UH, Tetens I, Hills AP, Michaelsen KF, Trolle E. Validation of a pre-coded food record for infants and young children. European journal of clinical nutrition. 2012;66(1):91–6. Epub 2011/08/11. doi: 10.1038/ejcn.2011.133 . 21829216 10.1038/ejcn.2011.133

[27] Fødevaredatabanken, version 7. [Internet]. Danmarks Tekniske Universitet. 2008. Available from: http://www.foodcomp.dk/.

[28] Gibson EL, Cooke L. Understanding Food Fussiness and Its Implications for Food Choice, Health, Weight and Interventions in Young Children: The Impact of Professor Jane Wardle. Current obesity reports. 2017;6(1):46–56. Epub 2017/02/17. doi: 10.1007/s13679-017-0248-9 . 28205158 10.1007/s13679-017-0248-9

[29] Smith AD, Herle M, Fildes A, Cooke L, Steinsbekk S, Llewellyn CH. Food fussiness and food neophobia share a common etiology in early childhood. Journal of child psychology and psychiatry, and allied disciplines. 2017;58(2):189–96. Epub 2016/10/16. doi: 10.1111/jcpp.12647 ; PubMed Central PMCID: PMCPMC5298015. 27739065 10.1111/jcpp.12647 PMC5298015

[30] Harris G, Coulthard H. Early Eating Behaviours and Food Acceptance Revisited: Breastfeeding and Introduction of Complementary Foods as Predictive of Food Acceptance. Current obesity reports. 2016;5(1):113–20. Epub 2016/03/10. doi: 10.1007/s13679-016-0202-2 ; PubMed Central PMCID: PMCPMC4796330. 26956951 10.1007/s13679-016-0202-2 PMC4796330

[31] Coulthard H, Harris G, Fogel A. Exposure to vegetable variety in infants weaned at different ages. Appetite. 2014;78:89–94. Epub 2014/04/02. doi: 10.1016/j.appet.2014.03.021 . 24685457 10.1016/j.appet.2014.03.021

[32] Forestell CA, Mennella JA. Early determinants of fruit and vegetable acceptance. Pediatrics. 2007;120(6):1247–54. Epub 2007/12/07. doi: 10.1542/peds.2007-0858 ; PubMed Central PMCID: PMCPMC2268898. 18055673 10.1542/peds.2007-0858 PMC2268898

[33] Husk JS, Keim SA. Breastfeeding and dietary variety among preterm children aged 1–3 years. Appetite. 2016;99:130–7. Epub 2016/01/23. doi: 10.1016/j.appet.2016.01.016 . 26792771 10.1016/j.appet.2016.01.016

[34] Burnier D, Dubois L, Girard M. Exclusive breastfeeding duration and later intake of vegetables in preschool children. European journal of clinical nutrition. 2011;65(2):196–202. Epub 2010/10/28. doi: 10.1038/ejcn.2010.238 . 20978527 10.1038/ejcn.2010.238

[35] Okubo H, Miyake Y, Sasaki S, Tanaka K, Hirota Y. Feeding practices in early life and later intake of fruit and vegetables among Japanese toddlers: the Osaka Maternal and Child Health Study. Public health nutrition. 2016;19(4):650–7. Epub 2015/06/04. doi: 10.1017/S1368980015001779 . 26036251 10.1017/S1368980015001779 PMC10270833

[36] Andrea SB, Hooker ER, Messer LC, Tandy T, Boone-Heinonen J. Does the association between early life growth and later obesity differ by race/ethnicity or socioeconomic status? A systematic review. Ann Epidemiol. 2017;27(9):583–92.e5. Epub 2017/09/16. doi: 10.1016/j.annepidem.2017.08.019 . 28911983 10.1016/j.annepidem.2017.08.019 PMC6688753

[37] Mitanchez D, Chavatte-Palmer P. Review shows that maternal obesity induces serious adverse neonatal effects and is associated with childhood obesity in their offspring. Acta Paediatr. 2018 Epub 2018/02/09. doi: 10.1111/apa.14269 . 29421859 10.1111/apa.14269

[38] Carducci B, Bhutta ZA. Care of the growth-restricted newborn. Best practice & research Clinical obstetrics & gynaecology. 2018;49:103–16. Epub 2018/03/25. doi: 10.1016/j.bpobgyn.2018.02.003 . 29571821 10.1016/j.bpobgyn.2018.02.003

[39] van Deutekom AW, Chinapaw MJ, Jansma EP, Vrijkotte TG, Gemke RJ. The Association of Birth Weight and Infant Growth with Energy Balance-Related Behavior—A Systematic Review and Best-Evidence Synthesis of Human Studies. PLoS One. 2017;12(1):e0168186 Epub 2017/01/13. doi: 10.1371/journal.pone.0168186 ; PubMed Central PMCID: PMCPMC5232347. 28081150 10.1371/journal.pone.0168186 PMC5232347

[40] Bruun S, Buhl S, Husby S, Jacobsen LN, Michaelsen KF, Sorensen J, et al Breastfeeding, Infant Formula, and Introduction to Complementary Foods-Comparing Data Obtained by Questionnaires and Health Visitors' Reports to Weekly Short Message Service Text Messages. Breastfeeding medicine: the official journal of the Academy of Breastfeeding Medicine. 2017;12(9):554–60. Epub 2017/08/24. doi: 10.1089/bfm.2017.0054 . 28832183 10.1089/bfm.2017.0054

[41] de Barse LM, Jansen PW, Edelson-Fries LR, Jaddoe VWV, Franco OH, Tiemeier H, et al Infant feeding and child fussy eating: The Generation R Study. Appetite. 2017;114:374–81. Epub 2017/04/13. doi: 10.1016/j.appet.2017.04.006 . 28400303 10.1016/j.appet.2017.04.006

[42] Miguel-Berges ML, Zachari K, Santaliestra-Pasias AM, Mouratidou T, Androutsos O, Iotova V, et al Clustering of energy balance-related behaviours and parental education in European preschool children: the ToyBox study. The British journal of nutrition. 2017;118(12):1089–96. Epub 2017/12/05. doi: 10.1017/S0007114517003129 . 29198192 10.1017/S0007114517003129

[43] dos Santos Silva I, Beral V. Socioeconomic differences in reproductive behaviour. IARC scientific publications. 1997;(138):285–308. Epub 1997/01/01. . 9353670

